# The Prognostic Role of Serum Procalcitonin for Adult Patients with Acute Diarrhea in the Emergency Department

**DOI:** 10.3390/diagnostics15060734

**Published:** 2025-03-15

**Authors:** Antonella Gallo, Marcello Covino, Eleonora Ianua’, Andrea Piccioni, Davide Della Polla, Benedetta Simeoni, Francesco Franceschi, Francesco Landi, Massimo Montalto

**Affiliations:** 1 Department of Geriatrics, Orthopedics and Rheumatology, Fondazione Policlinico Universitario “A. Gemelli”, IRCCS, 00168 Rome, Italy; antonella.gallo@policlinicogemelli.it (A.G.); francesco.landi@unicatt.it (F.L.); massimo.montalto@unicatt.it (M.M.); 2 Department of Emergency Medicine, Fondazione Policlinico Universitario “A. Gemelli”, IRCCS, 00168 Rome, Italy; andrea.piccioni@policlinicogemelli.it (A.P.); davide.dellapolla@policlinicogemelli.it (D.D.P.); benedetta.simeoni@policlinicogemelli.it (B.S.); or francesco.franceschi@unicatt.it (F.F.); 3 Department of Emergency Medicine, Università Cattolica del Sacro Cuore, 00168 Roma, Italy; 4 Department of Internal Medicine, Università Cattolica del Sacro Cuore, 00168 Roma, Italy; eleonora.ianua01@icatt.it; 5 Department of Geriatrics, Orthopedics and Rheumatology, Università Cattolica del Sacro Cuore, 00168 Roma, Italy

**Keywords:** acute diarrhea, emergency department, procalcitonin, biomarkers

## Abstract

**Background.** Acute diarrhea is one of the leading causes of Emergency Department (ED) access. The search for the causative pathogen cannot be routinely performed since conventional methods, like stool cultures, are time-consuming, requiring days for growth and delaying diagnosis and the start of therapy. In this large sample retrospective study, we evaluated the prognostic role of serum procalcitonin (PCT) for adult patients with acute diarrhea in the ED. **Methods.** In a retrospective, mono-centric study, we enrolled all patients visiting our ED complaining of acute diarrhea and then hospitalized over five years. Final diagnosis of an infective (including bacterial) diarrhea, any other infection, and a bloodstream infection (BSI) was collected by clinical records, according to the International Disease Classification 10th edition. Procalcitonin determination was obtained upon request of the ED physician at the admission visit based on patient evaluation and clinical judgment. **Results.** Of a total of 1910 patients, early PCT values (cut-off of 0.5 ng/mL) did not show a significant predictive value for infective diarrhea (OR 0.554 [0.395–0.778]), nor for bacterial diarrhea (OR 0.596 [0.405–0.875]). Conversely, PCT levels at ED admission showed a significant predictive value for a final diagnosis of any infection (OR 1.793 [1.362–2.362]) and, above all, of bloodstream infection (BSI) (OR 6.694 [4.869–9.202]). **Conclusions.** Our data suggest that in ED, where the complexity and heterogeneity of patients are very high, indiscriminate PCT-guided management of patients with diarrhea is not indicated. Conversely, in patients with diarrhea but also clinical suspicion of BSI, PCT determination remains a useful instrument, possibly improving clinical management.

## 1. Introduction

Acute diarrhea represents a leading cause of mortality and morbidity worldwide [1] and is one of the most common symptoms reported by patients referred to the Emergency Department (ED) [1,2]. Even in the most developed countries, it is often of infectious origin, mainly viral [3]; however, looking for diarrhea etiology usually represents a challenge for the ED physician, as it may be present in various clinical conditions [4].

Since now, only a few studies focused on acute diarrhea etiology among adults visiting the ED [3,4,5,6]. Viruses, mainly Norovirus, resulted as the most frequently involved agents, above all during the winter months [3,4]. Bacterial infections, mainly by *Salmonella* and *Vibrio parahaemolyticus* agents, were responsible for about a quarter of cases. However, in more than one-third of cases, the etiological agent was not detected [3,4].

As a result, providing an accurate diagnosis based solely on the clinical presentation in the ED could be difficult, and when infectious diarrhea is suspected, the early diagnosis of acute bacterial gastroenteritis remains challenging. Laboratory investigation in healthy and immunocompetent subjects is usually not indicated considering that the majority of acute diarrheal illnesses are often of short duration, self-limited, or characterized by mild symptoms [6,7,8]. However, selected patients, above all frailer and immunosuppressed patients, may deserve a more accurate diagnostic work-up since the higher risk of complications and severe disease is usually associated with specific pathogens [6,7,9].

An early and accurate diagnosis can significantly contribute to identifying the correct strategy, guiding physicians in the decision-making process, supporting in the discrimination of those patients that can be safely discharged to home or need hospitalization, and, in particular, aiding in the decision whether to prescribe an empiric antibiotic therapy. Nevertheless, the search for the causative pathogen cannot be routinely performed in the ED since conventional methods, like stool culture, are time-consuming, requiring days for growth and delaying diagnosis and consequent therapeutic approach [10,11].

Non-invasive techniques, including stool or blood biomarkers, may represent feasible and reliable tools able to improve clinical management. To date, however, only a few studies have been focused on this topic [12,13,14,15]. In the last decade, interest has grown in real-time PCR-based multiplex stool testing, able to provide a result within one hour, and, consequently, early identification of the main viral, bacterial, and parasitic pathogens responsible for acute diarrhea [16,17]. However, its availability is still limited and is not routinely performed in an ED setting.

Serum procalcitonin (PCT) is a precursor protein of calcitonin expressed by human cells [18]. Its production is upregulated by pro-inflammatory cytokines, like interleukin (IL)-1, IL-2, IL-6, tumor necrosis factor-alpha, and by bacterial endotoxins and lipopolysaccharide. Additionally, it is downregulated during viral infections [18,19]. Procalcitonin determination has been recently approved in acute respiratory infections, providing good predictive values for the diagnosis and exclusion of pneumonia, and, above all, for a diagnosis of sepsis [20]. Different works show that it can perform better than C-reactive protein (CRP) for bacterial infection among inpatients [14,20] and can also improve clinical outcomes [21,22]. In particular, PCT-guided management, mainly regarding antibiotic therapy, leads to a significant benefit in patient management, showing the best advantage in high-risk patient populations [23]. Conversely, in a heterogeneous population, such as non-septic ED patients with fever [24] or those affected by lower respiratory tract [25] or urinary tract infections [26], current evidence is not conclusive.

To date, no definitive data are available about the usefulness of PCT in adult ED patients with diarrhea [13,14,15]. This study aimed to clarify the prognostic role of an early PCT determination in the management of adult patients with acute diarrhea in the ED.

## 2. Materials and Methods

This is a retrospective mono-centric study conducted in a tertiary urban University Hospital with annual attendance at the ED of about 75,000 patients (more than 87% adults). We evaluated the clinical records of consecutive patients admitted to our ED in five years from 1 January 2018 to 31 December 2023. We included all patients accessing the ED for acute diarrhea in the 24 h before the admission who had a PCT determination in the ED and were subsequently hospitalized. We excluded from the study cohort patients < 18 years old and all patients discharged home or transferred to other hospitals from the ED.

### 2.1. Patient Characteristics and Clinical History

All clinical and demographic data were extracted anonymously from the hospital’s computerized clinical records. Patient data were reviewed to assess demographic data, major comorbidities, and clinical symptoms at admission.

All patients included in the study cohort were evaluated for the following:

- Demographic data (age, sex).
- Vital signs at the ED admission (arterial pressure, heart rate, body temperature).
- Clinical symptoms (including cough, chest pain, abdominal pain, vomiting, gastrointestinal bleeding, neurological impairment, renal failure, asthenia).
- Comorbidities: We recorded the comorbidities included in the Charlson’s comorbidity score which was calculated for all the patients [27]. In particular, history of cancer, solid and hematological transplantation, inflammatory bowel disease (IBD), ischemic cardiac disease, heart failure, chronic obstructive pulmonary disease (COPD), diabetes mellitus, chronic renal failure, liver disease, and dementia were collected from clinical records.
- Laboratory findings included hemoglobin, white cell count, fibrinogen glucose, platelet count, procalcitonin, C-reactive protein, and serum creatinine. The procalcitonin determination was obtained upon request of the emergency physician based on patient evaluation and clinical judgment. Procalcitonin determinations were available 24 h a day in our ED, with a lab time response of about 1 h.
- Clinical outcomes were evaluated at hospital discharge or death and included all-cause in-hospital death, length of hospital stay (LOS), need for abdominal surgery, diagnosis of infective gastroenteritis (either bacterial or viral), diagnosis of bloodstream infection, diagnosis of any infective disease.

### 2.2. Outcome Measures

The definition of infective diarrhea included “any or specified bacterial diarrhea, including *Clostridioides difficile* infection”, and “probable infective diarrhea”. The other infective diagnosis included “any other infection (respiratory infection, urinary tract infection, abdominal infection, and other types of infection if not included in the previous ones)” and bloodstream infection (BSI). The diagnoses were based on the clinical records according to the International Disease Classification 10th edition (ICD-10) [28].

The in-hospital length of stay (LOS) was calculated from the time of ED admission to the hospital discharge.

### 2.3. Study Endpoints

As the primary endpoint, we evaluated the role of serum PCT in the diagnosis of acute gastrointestinal infection.

As secondary endpoints, we evaluated the relationship between serum PCT and diagnosis of bloodstream infection, the diagnosis of any infective disease, and in-hospital death.

### 2.4. Statistical Analysis and Sample Size

Categorical variables are presented as absolute numbers and percentages; continuous variables are presented as median (interquartile range). Categorical variables were statistically compared using Chi-square test or Fisher exact test, as appropriate. Continuous variables were compared with the Mann–Whitney U test or, in the case of three or more groups, with the Wilcoxon ANOVA median test.

Significant factors at univariate analysis were entered into a multivariate logistic regression model to identify independent risk predictors for the defined outcomes. The significant variables at univariate analysis were included in a logistic model to find the independent predictors for the defined outcomes. In the case of combined variables, such as single comorbidities and the Charlson Index, the composing factors were excluded from the analysis to avoid parameter overestimation and instability. The finding of an abnormal PCT value (>0.5 ng/mL) was forced in all the logistic models. Similarly, to include in the analysis the overall burden of comorbidities for each patient, the Charlson Index was forced in all the analyses. Logistic regression analysis results were reported as Odds Ratio (OR) [95% confidence interval].

The discrimination ability of serum Procalcitonin for the defined outcomes was evaluated with the Receiver Operating Characteristics (ROC) curve analysis. Survival curves were calculated using the Kaplan–Meier method. All p values were 2-sided, with a significance threshold set at 0.05, and corrected in case of multiple group comparison.

No a priori sample size calculation was performed because all eligible records were used in this retrospective study

The study analysis was conducted with SPSS version 25 (IBM, Armonk, NY, USA).

### 2.5. Statement of Ethics

The study was conducted according to the principles expressed in the Declaration of Helsinki and its later amendments and approved by the Institutional Review Board (#0025817/22 on 03/08/2022).

The authors declare no conflict of interest.

## 3. Results

During the study period, 2125 patients evaluated in the ED for acute diarrhea were admitted to our hospital wards. Among these, 215 did not meet inclusion criteria or had incomplete or inconsistent clinical records and were excluded. Thus, the study cohort consisted of 1910 patients with a median age of 70 [55–80] years. There were 893 (46.8%) males. Early PCT determination at ED admission resulted below the normal range (<0.5 ng/mL) in 1528 (65.9%) patients and above the normal range in 649 (34.1%) patients. Other clinical and demographic data of enrolled patients are shown in Table 1.

**Figure 1:**
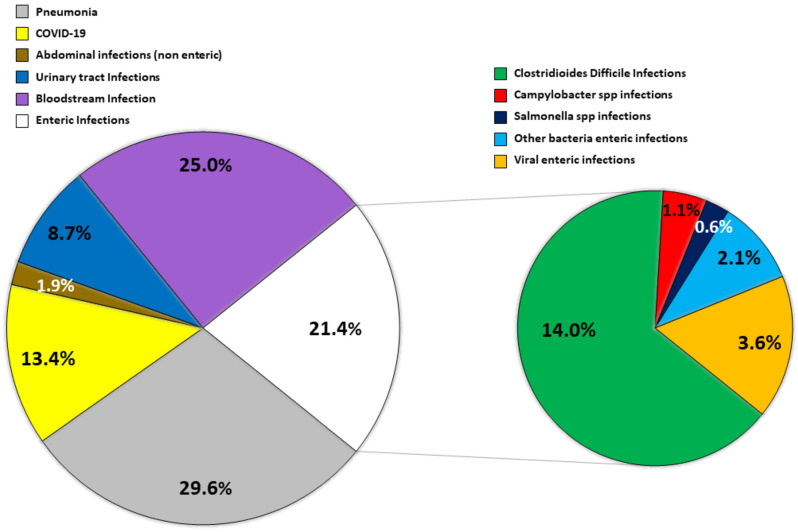
Infective diagnosis at discharge in the total population.

Figure 1 shows the distribution of infective diagnosis at hospital discharge, with a particular focus on those patients receiving a definitive infective bacterial diagnosis, as confirmed by stool cultural testing.

In-hospital death occurred in 207 cases (10.8%), and the median LOS was 9.0 [5.7–15.3] days. ICU admission occurred in 89 (4.7%) patients. As expected, the LOS, the in-hospital death, and the percentage of ICU admission were higher in those subjects showing increased values of PCT at ED admission (Table 1). Abdominal surgery was performed in 101 (5.5%) patients; in this case, no significant differences were reported according to PCT values at ED admission.

### 3.1. Early PCT Determination in Infective Intestinal Diagnosis

At discharge, infective diarrhea was reported in 285 (14.9%) patients in the study cohort. Table 2 shows differences among these subjects and those not receiving this final diagnosis. At univariate analysis, PCT values at ED admission did not correlate with a final infective diagnosis (*p* = 0.813).

At multivariate analysis, early PCT values (with a cut-off of 0.5 ng/mL) did not discriminate between the two groups (OR 0.554 [0.395–0.778]), conversely to white blood cell (WBC) count (OR 1.015 [1.002–1.028]), serum creatinine levels (OR 1.020 [0.919–1.097]), and C-reactive protein (CRP) levels (OR 1.001 [1.000–1.003]). Moreover, older (OR 1.025 [1.014–1.037]) and more comorbid patients (OR 1.004 [0.949–1.076]) more likely received a final intestinal infective diagnosis.

These results were confirmed when analyzing the subgroups of patients receiving a final diagnosis of bacterial diarrhea (Table 3).

Also, in these patients, univariate analysis showed that PCT values at ED admission did not correlate with a final diagnosis of bacteria diarrhea (*p* = 0.430). As the same, at multivariate analysis, early PCT values did not discriminate between the two groups (OR 0.596 [0.405–0.875]), conversely to white blood cell (WBC) count (OR 1.026 [1.010–1.043]), serum creatinine levels (OR 1.004 [0.917–1.098]), and C-reactive protein (CRP) levels (OR 1.002 [1.000–1.004]). Also, in this case, older (OR 1.042 [1.024–1.057]) and more comorbid patients (OR 1.005 [0.926–1.076]), in addition to febrile patients (OR 1.043 [0.736–1.477]), more likely received a final diagnosis of bacteria diarrhea.

### 3.2. Early PCT Determination in Non-Intestinal Infection Diagnosis

Table 4 and Table 5 report the association of the same parameters with a final diagnosis of any infection (not including intestinal infection) and BSI.

Conversely to intestinal infective diagnosis and bacteria diarrhea, PCT levels at ED admission showed the strongest predictive value for a final diagnosis of any infection (OR 1.793 [1.362–2.362]), performing better than WBC count (OR 1.009 [0.999–1.020]) and CRP levels (OR 1.003 [1.001–1.004]). These data resulted as more significant for a final diagnosis of BSI, as shown in Table 5.

In this case, PCT levels at ED admission showed the strongest predictive value (OR 6.694 [4.869–9.202]), performing much better than WBC count (OR 1.002 [0.989–1.015]) and CRP levels (OR 1.002 [1.000–1.003]).

In each of the two subgroups, older patients were more likely to receive an infective diagnosis or a diagnosis of BSI.

## 4. Discussion

This represents the first retrospective study on a large sample aimed at evaluating the usefulness of early PCT determination in a heterogeneous real-world ED adult population hospitalized for diarrhea.

Our main finding is that PCT determination at ED admission does not represent a significant predictive biomarker for a final infective intestinal diagnosis, even of bacterial etiology.

Management of patients with acute diarrhea arriving at the ED often represents a challenge for physicians. An early discrimination between infectious or non-infectious etiology, and between bacterial and viral origin, could ideally guide different therapeutic approaches.

Current guidelines, provided by the American College of Gastroenterology (ACG) [7] and the Infectious Diseases Society of America (IDSA) Society [8] represent the most recent recommendations on the management of adults with acute diarrhea, although there are no specific paragraphs dedicated to the ED setting.

While, regardless of the etiology, there is a consensus about common measures such as fluid repletion and nutrition maintenance that should be promptly warranted to all subjects, the major concerns regard antibiotic prescription. Emergency physicians notoriously have to deal both with limited information to achieve a definitive diagnosis and with patients’ expectations and satisfaction, already known to possibly influence the therapeutical approach [29]. Karras et al. showed that, in a cohort of American adults and children admitted to the ED with diarrhea, physicians more likely prescribed antibiotics not only for suspicion of bacterial enteritis but also when they simply believed that patients expected to be given them [29]. Moreover, whereas 100% of patients treated with antibiotics were satisfied with their treatment, only 90% of patients who were not prescribed antibiotics showed the same level of satisfaction [29].

Since etiologic diagnosis by stool culture or polymerase chain reaction requires time and costs, often incompatible with the ED setting, the search for accurate, rapid, and non-invasive diagnostic tools can, therefore, represent one of the most interesting focuses of research in the management of diarrheal diseases. However, only a few small sample studies are available on this topic, mainly conducted in the children population [12,13,14,15,30,31].

Among different available inflammatory biomarkers, interest in the role of PCT determination in a particular clinical setting such as the ED has been rapidly growing. Levels of PCT increase significantly and early when an inflammatory bacterial process occurs, due to inflammatory cytokines and bacterial endotoxins being released [18,19,20]. A PCT-guided approach has been largely reported to significantly and positively impact overall patient management in different infectious conditions, most of all in BSI [21,22,23,24,25,26].

Nevertheless, conclusive evidence on the utility of PCT in patients complaining of acute diarrhea is still lacking [12,13,14,15]. Thia et al. [13] performed a prospective study on 81 patients with acute gastroenteritis (as confirmed by stool cultures for standard pathogens), 18.5% of whom were of bacterial etiology, showing that levels of PCT and C-reactive protein helped in the discrimination of bacterial and undifferentiated gastroenteritis. However, the sample of this study was very small since only fifteen patients received a diagnosis of bacterial gastroenteritis [13].

Also, in a pediatric setting [30], Ismaili-Jaha et al. found that PCT represented an important but not conclusive marker of bacterial etiology in children younger than 5 years since its levels resulted in a significant difference between bacterial and viral gastroenteritis, but they were not able to discriminate between bacterial and extra-intestinal diarrhea [30].

More recently, Shin et al. [14] reported in a cohort of 514 patients with diarrhea that the determination of serum PCT may have a significant predictive value (OR 1.321, AUC 0.797) for the detection of inflammatory diarrhea, showing a better predictive value compared with CRP (OR 1.145, AUC 0.697). Discrimination between inflammatory and non-inflammatory group diarrhea was based on endoscopic and/or radiological parameters, such as abnormal bowel wall thickening and fluid collection at abdominal CT or typical mucosal alterations on colonoscopy [14].

In this retrospective study, all patients were supposed to be affected by “acute infectious diarrhea”; although no stool cultural examinations or discharge diagnosis were provided, general inclusion criteria consisted of fever (>37.8 °C), abdominal pain, and diarrhea. Similarly, no information about the clinical history and comorbidities were reported. It is plausible that higher levels of PCT may be related to the intensity of the inflammatory response, maybe identifying more severe clinical pictures and potentially helping the clinician’s decision-making in starting or not starting an empiric antibiotic treatment. However, as the same authors concluded, a global patient evaluation should be encouraged always when approaching patients with acute diarrhea and PCT levels alone is not a gold standard for the inflammatory subgroup [14].

As average age increases, concepts such as frailty, comorbidity, and functional status have been receiving growing attention to improve health care in a population notably experiencing higher rates of adverse outcomes compared to other people of the same age [32,33,34]. Therefore, a global assessment of frailty and functional status should be encouraged and incorporated into the standard evaluation, regardless of age, including in people visiting the ED for acute diarrhea [6,34]. An early and careful identification of the most vulnerable patients, in fact, may significantly support physicians in targeting the approach and balancing the beneficial and harmful potential of each intervention [32,33,34].

Our results showed that an indiscriminate PCT sampling in all ED populations complaining of acute diarrhea should not be recommended; rather it should be reserved for those subjects with clinical features suggestive for sepsis associated with diarrhea. This result confirmed both our previous findings showing that a PCT-guided management in ED results in benefits only in severely ill patients [24]. Conversely, in more heterogeneous populations, even if admitted to ICU wards, the PCT-guided management did not show the same significant advantages compared to standard management [26,35].

In particular, we found that early PCT determination at ED admission cannot predict a bacterial infectious diarrhea diagnosis, even a *Clostridioides difficile* infection. This result is in line with a previous but smaller study by Casella de Abreu et al. [15]. They included 80 patients admitted to the ED with acute infectious diarrhea, 21 with acute colitis and 24 with another illness causing diarrhea. They found that PCT levels remained low in the case of bacterial enteritis, concluding that this biomarker cannot represent a guide to antibiotic therapy in these patients [15].

It is known that CRP is the most frequent biomarker used in the ED in patients admitted for acute diarrhea [36]. In our study, as for de Abreu et al. [15], CRP levels showed a better predictive value than PCT, although the lack of widely recognized cut-offs limits its utility in discriminating between bacterial and viral infections, thus not helping physicians in deciding whether or not to start an antibiotic therapy [14]. Molecular testing, like PCR GI panel, may be very useful in the ED setting, due to the quickness of results (about 1 h) and the high diagnostic accuracy in pathogen detection [16,17]. However, the accessibility to these diagnostic tools is still limited, and further studies are needed to confirm their usefulness in clinical practice.

### 4.1. Study Limitations

Although conducted on a very large cohort of patients, our study had some limitations: first, its retrospective nature. For this reason, we were not able to retrieve the information about the use of antibiotic therapy in the month before the ED access. Moreover, the final discharge diagnosis was collected by reviewing clinical records, and a comparison with a gold standard such as stool or blood culture examinations was not available for most of the patients. Moreover, we only measured PCT levels on ED admission and did not collect the following levels during admission. However, our main purpose was to evaluate the usefulness of the early measurement on ED admission and not during hospitalization, when, usually, physicians have more time and more accurate diagnostic tests available to guide them in identifying the correct approach.

### 4.2. Conclusions

Our data suggest that in ED, where the complexity and heterogeneity of patients are very high, indiscriminate PCT-guided management of patients with diarrhea is not indicated. Conversely, in patients with diarrhea and a clinical suspicion of BSI, PCT determination remains a useful instrument, possibly improving clinical management.

## Figures and Tables

**Table 1:** Demographic and clinical characteristics of the 1910 patients included in the study.

*Variable*		*PCT Values*		*Univ.*
		*<0.5 (n. 1258)*	*>0.5 (n. 649)*	*p Values*
Males	893 (46.8%)	583 (46.3%)	310 (47.5%)	0.617
Median age (years)	70 [55–80]	69 [53–80]	71 [59–80]	0.008
***ED Presentation***				
Triage Code				<0.001
- Emergency	97 (5.1%)	42 (3.3)	55 (8.4)	
- Urgent	528 (27.6%)	308 (24.5)	220 (33.7)	
- Non-urgent	1285 (67.3%)	908 (72.2)	377 (57.8)	
***Associated symptoms***				
Cough	193 (10.1)	148 (11.8)	45 (6.9)	0.001
Vomiting	636 (33.3)	429 (34.1)	207 (31.7)	0.301
Abdominal pain	716 (37.5)	485 (38.6)	231 (35.4)	0.181
Chest pain	49 (2.6)	35 (2.8)	14 (2.1)	0.405
Syncope	188 (9.8)	117 (9.3)	71 (10.9)	0.269
Gastrointestinal bleeding	101 (5.3)	76 (6.0)	25 (3.8)	0.041
Asthenia	386 (20.2)	244 (19.4)	142 (21.8)	0.219
Confusion	112 (5.9)	66 (5.2)	46 (7.1)	0.111
Anuresis	35 (1.8)	15 (1.2)	20 (3.1)	0.004
***Laboratory parameters***				
Hb (g/dL)	12.2 [10.4–13.7]	12.5 [10.7–13.9]	11.7 [10.0–13.3]	<0.001
WBC (cell/mm^3^)	9.9 [6.5–14.7]	9.1 [6.4–12.8]	12.4 [7.2–17.8]	<0.001
Creatinine (mg/dL)	1.0 [0.8–1.6]	0.9 [0.7–1.3]	1.4 [0.9–2.4]	<0.001
Blood glucose (mg/dL)	111.0 [96.0–140.0]	109.0 [96.0–133.0]	118.0 [99.0–150.5]	<0.001
Procalcitonin (ng/dL)	0.19 [0.06–1.01]	0.09 [0.05–0.18]	2.60 [0.97–13.9]	<0.001
CRP (mg/L)	81.5 [25.7–156.0]	47.1 [15.7–115.6]	156 [88.1–230.2]	<0.001
***Clinical history***				
Charlson comorbidity score	4 [2–6]	4 [2–6]	5 [3–6]	<0.001
Cancer	378 (19.8)	220 (17.5)	158 (24.2)	<0.001
Immunosuppressive therapy	54 (2.8)	37 (2.9)	17 (2.6)	0.676
IBD	136 (7.1)	117 (9.3)	19 (2.9)	<0.001
Ischemic cardiac disease	174 (9.1)	108 (8.6)	66 (10.1)	0.268
Heart failure	123 (6.4)	86 (6.8)	37 (5.7)	0.327
COPD	150 (7.9)	99 (7.9)	51 (7.8)	0.971
Diabetes	237 (12.4)	139 (11.0)	98 (15.0)	0.012
Chronic renal failure	330 (17.3)	161 (12.8)	169 (25.9)	<0.001
Liver disease	145 (7.6)	92 (7.3)	53 (8.1)	0.523
Dementia	147 (7.7)	101 (8.0)	46 (7.1)	0.449
***Main Infective discharge diagnosis***				
Bacterial diarrhea	207 (10.8)	142 (11.3)	65 (10.0)	0.379
Infective diarrhea (any)	285 (14.9)	202 (16.1)	83 (12.7)	0.053
Infection (any)	1248 (65.3)	753 (59.9)	495 (75.9)	<0.001
BSI	337 (17.8)	92 (7.3)	245 (37.6)	<0.001
COVID-19 infection	179 (9.4)	150 (11.9)	29 (4.4)	<0.001
***Clinical outcomes***				
Lenght of hospitalization	9.0 [5.7–15.3]	8.4 [5.5–14.4]	10.0 [6.0–17.2]	0.002
Death	207 (10.8)	100 (7.9)	107 (16.4)	<0.001
ICU admission	89 (4.7)	35 (2.8)	54 (8.3)	<0.001
Abdominal surgery	101 (5.3)	67 (5.3)	34 (5.2)	0.918

**Table 2:** Demographic and clinical features of patients with a final diagnosis of infective diarrhea.

*Variable*	*Infecrive Diarrhea*		*Univ.* *p Value*	*Odds Ratio * *[95% CI]*	*Multiv. * *p Value*
	*NO (N 1625)*	*YES (N 285)*			
Sex (Male)	778 (47.9%)	115 (40.4%)	**0.019**	0.800 [0.613–1.045]	0.101
Median age (years)	69 [54–79]	78 [66–83]	**<0.001**	1.023 [1.013–1.034]	<0.001
***ED Presentation***					
Triage Code			0.425		
- Emergency	85 (5.2)	12 (4.2)			
- Urgent	456 (28.1)	72 (25.3)			
- Non-urgent	1084 (66.7)	201 (70.5)			
***Associated symptoms***					
Cough	184 (11.3)	9 (3.2)	**0.001**	0.265 [0.133–0.529]	<0.001
Vomiting	542 (33.4)	94 (33.0)	0.902		
Abdominal pain	626 (38.5)	90 (31.6)	0.026		
Chest pain	44 (2.7)	5 (1.8)	0.348		
Syncope	162 (10.0)	26 (9.1)	0.658		
Gastrointestinal bleeding	86 (5.3)	15 (5.3)	0.964		
Asthenia	340 (20.9)	46 (16.1)	0.064		
Confusion	96 (5.9)	16 (5.6)	0.846		
Anuresis	32 (2.0)	3 (1.1)	0.287		
***Laboratory parameters***					
Hb (g/dL)	12.2 [10.4–13.8]	12.1 [10.4–13.5]	0.566		
WBC (cell/mm^3^)	9.6 [6.3–14.3]	11.0 [7.6–16.4]	**<0.001**	1.015 [1.002–1.028]	0.027
Creatinine (mg/dL)	0.9 [0.7–1.3]	1.4 [0.9–2.4]	**0.018**	1.040 [0.974–1.111]	0.244
Blood glucose (mg/dL)	112.0 [97.0–140.7]	109.0 [94.5–135.5]	0.151		
PCT (ng/dL)	0.18 [0.06–1.09]	0.21 [0.07–13.9]	0.813		
PCT > 0.5 ng/dL (%)	569 (35.0)	83 (29.1)	0.053	0.629 [0.470–0.843]	0.002
CRP (mg/L)	77.8 [24.6–156.2]	101.4 [38.3–153.9]	0.054		
***Clinical history***					
Charlson comorbidity score	4 [2–6]	5 [3–6]	**<0.001**	0.966 [0.906–1.029]	0.278
Immunosuppressive therapy	48 (3.0)	8 (2.1)	0.425		
IBD	129 (7.9)	7 (2.5)	**0.001**	0.336 [0.152–0.740]	0.007
Ischemic cardiac disease	149 (9.2)	25 (8.8)	0.830		
Heart failure	93 (5.7)	30 (10.5)	0.002		
COPD	123 (7.6)	27 (9.5)	0.270		
Diabetes	196 (12.1)	41 (14.4)	0.272		
Chronic renal failure	265 (16.3)	65 (22.8)	0.007		
Liver disease	130 (8.0)	15 (5.3)	0.108		
Dementia	116 (7.1)	31 (10.9)	0.0.29		
Cancer	349 (21.5)	29 (10.2)	<0.001		
***Clinical outcomes***					
Abdominal surgery	95 (5.8)	8 (2.1)	0.009		
Lenght of hospitalization	8.9 [5.5–15.0]	9.4 [6.0–15.4]	0.279		

**Table 3:** Demographic and clinical features of patients with a final diagnosis of bacterial diarrhea.

*Variable*	*Bacterial Diarrhea*		*Univ.* *p Value*	*Odds Ratio* *[95% CI]*	*Multiv.* *p Value*
	*NO (N 1703)*	*YES (N 207)*			
Sex (Male)	815 (47.9%)	78 (37.7%)	**0.006**	0.728 [0.527–1.005]	0.054
Median age (years)	69 [54–79]	79 [71–84]	**<0.001**	1.045 [1.031–1.059]	<0.001
***ED Presentation***					
Triage Code			0.562		
- Emergency	87 (5.1)	10 (4.8)			
- Urgent	477 (28.0)	51 (24.6)			
- Non-urgent	1139 (66.9)	146 (70.5)			
***Associated symptoms***					
Cough	187 (11.0)	6 (2.9)	**<0.001**	0.274 [0.118–0.638]	0.003
Vomiting	574 (33.7)	62 (30.0)	0.279		
Abdominal pain	653 (38.3)	63 (30.4)	**0.026**	0.796 [0.569–1.114]	0.183
Chest pain	46 (2.7)	3 (1.4)	0.282		
Syncope	170 (10.0)	18 (8.7)	0.557		
Gastrointestinal bleeding	94 (5.5)	7 (3.4)	0.194		
Asthenia	354 (20.8)	32 (15.5)	0.071		
Confusion	100 (5.9)	12 (5.8)	0.965		
Anuresis	33 (1.9)	2 (1.0)	0.325		
***Laboratory parameters***					
Hb (g/dL)	12.3 [10.5–13.8]	11.7 [10.3–13.2]	**0.022**	0.988 [0.921–1.060]	0.739
WBC (cell/mm^3^)	9.6 [6.3–14.2]	12.3 [8.3–18.1]	**<0.001**	1.023 [1.008–1.038]	0.003
Creatinine (mg/dL)	1.0 [0.8–1.6]	1.1 [0.7–2.0]	0.103		
Blood glucose (mg/dL)	111.0 [97.0–140.0]	113.0 [94.0–137.0]	0.434		
PCT (ng/dL)	0.18 [0.06–1.06]	0.21 [0.08–0.78]	0.430		
PCT > 0.5 ng/dL (%)	587 (34.5)	65 (31.4)	0.379	0.617 [0.420–0.906]	0.014
CRP (mg/L)	75.7 [24.8–154.1]	116.7 [45.1–160.8]	**0.002**	1.002 [1.000–1.004]	0.024
***Clinical history***					
Charlson comorbidity score	4 [2–6]	5 [4–6]	**<0.001**	0.960 [0.889–1.037]	0.303
Immunosuppressive therapy	51 (3.0)	3 (1.4)	0.205		
IBD	130 (7.6)	6 (2.9)	**0.012**	0.483 [0.189–1.231]	0.127
Ischemic cardiac disease	157 (9.2)	17 (8.2)	0.635		
Heart failure	102 (6.0)	21 (10.1)	0.021		
COPD	127 (7.5)	23 (11.1)	0.065		
Diabetes	208 (12.2)	29 (14.0)	0.459		
Chronic renal failure	280 (16.4)	50 (24.2)	0.006		
Liver disease	133 (7.8)	12 (5.8)	0.302		
Dementia	120 (7.0)	27 (13.0)	0.002		
Cancer	359 (21.1)	19 (9.2)	<0.001		
***Clinical outcomes***					
Need for abdominal surgery	95 (5.6)	6 (2.9)	0.104		
Lenght of hospitalization	8.5 [5.5–15.0]	11–0 [7.2–19.0]	<0.001		

**Table 4:** Demographic and clinical features of patients with a final diagnosis of any infection.

*Variable*	*Infection (Any)*		*Univ.* *p Value*	*Odds Ratio* *[95% CI]*	*Multiv.* *p Value*
	*NO (N 662)*	*YES (N 1248)*			
Males	309 (46.7%)	584 (46.8%)	0.961		
Median age (years)	66 [52–78]	72 [58–82]	**<0.001**	1.030 [1.022–1.038]	<0.001
***Ed Presentation***					
Triage Code			0.372		
- Emergency	32 (4.8)	65 (5.2)			
- Urgent	196 (29.6)	332 (26.6)			
- Non-urgent	434 (65.6)	851 (68.2)			
*Vital signs*					
Heart rate	90 [78–105]	90 [79–104]	0.967		
Maximum Blood pressure (mmHg)	120 [103–139]	120 [103–139]	0.837		
Fever (>37.5 °C)	421 (63.6)	984 (78.8)	<0.001		
***Associated symptoms***					
Cough	42 (6.3)	151 (12.1)	**<0.001**	1.888 [1.280–2.784]	0.001
Vomiting	221 (33.4)	415 (33.3)	0.954		
Abdominal pain	293 (44.3)	423 (33.9)	**<0.001**	0.775 [0.624–0.962]	0.021
Chest pain	22 (3.3)	27 (2.2)	0.127		
Syncope	65 (9.8)	123 (9.9)	0.979		
Gastrointestinal bleeding	56 (8.5)	46 (3.6)	**<0.001**	0.539 [0.344–0.844]	0.007
Asthenia	151 (22.8)	235 (18.8)	**0.039**		
Confusion	36 (5.4)	76 (6.1)	0.563		
Anuresis	15 (2.3)	20 (1.6)	0.304		
**Laboratory parameters**					
Hb (g/dL)	12.2 [10.3–13.8]	12.2 [10.5–13.7]	0.392		
WBC (cell/mm^3^)	9.5 [6.5–13.5]	10.1 [6.6–13.5]	**0.005**	1.006 [0.994–1.017]	0.352
Creatinine (mg/dL)	1.0 [0.7–1.7]	1.0 [0.8–1.6]	0.087		
Blood glucose (mg/dL)	111.0 [96.0–138]	111.0 [97.0–141.0]	0.495		
PCT (ng/dL)	0.14 [0.05–0.47]	0.25 [0.07–1.68]	<0.001		
PCT > 0.5 ng/dL (%)	157 (23.7)	495 (39.7)	**<0.001**	1.634 [1.258–2.121]	<0.001
CRP (mg/L)	50.8 [12.5–129.5]	95.9 [38.3–171.4]	**<0.001**	1.003 [1.002–1.004]	<0.001
**Clinical history**					
Charlson comorbidity score	4 [2–6]	5 [3–6]	0.125	0.859 [0.819–0.902]	<0.001
Immunosuppressive therapy	18 (2.7)	36 (2.9)	0.835		
IBD	99 (15.0)	37 (3.0)	<0.001	0.234 [0.153–0.360]	<0.001
Ischemic cardiac disease	53 (8.0)	121 (9.7)	0.222		
Heart failure	39 (5.9)	84 (6.7)	0.477		
COPD	24 (3.6)	126 (10.1)	<0.001		
Diabetes	83 (12.5)	154 (12.3)	0.272		
Chronic renal failure	107 (16.2)	223 (17.9)	0.348		
Liver disease	60 (9.1)	85 (6.8)	0.077		
Dementia	32 (4.8)	115 (9.2)	0.001		
Cancer	192 (29.0)	186 (14.9)	<0.001		
***Clinical outcomes***					
Abdominal surgery	25 (3.8)	76 (6.1)	0.032		
Lenght of hospitalization	7.5 [5.1–12.4]	10.0 [6.0–17.0]	<0.001		

**Table 5:** Demographic and clinical features of patients with a final diagnosis of sepsis.

*Variable*	*Sepsis*		*Univ. * *p Value*	*Odds Ratio * *[95% CI]*	*Multiv. * *p Value*
	*NO (N 1573)*	*YES (N 337)*			
Males	734 (46.7%)	159 (47.2%)	0.863	1.011 [0.768–1.329]	0.940
Median age (years)	69 [54–80]	73 [62–81.5]	<0.001	1.008 [0.997–1.019]	0.152
***ED presentation***					
Triage Code			<0.001		
- Emergency	57 (3.6)	40 (11.9)			
- Urgent	401 (25.5)	127 (37.7)			
- Non-urgent	1115 (70.9)	170 (50.4)			
*Vital signs*					
Heart rate	89 [78–104]	94 [82.3–94]	0.001		
Maximum Blood pressure (mmHg)	121 [106–139]	113 [95–136]	<0.001		
Fever (>37.5 °C)	1152 (73.2)	253 (75.1)	0.487	0.960 [0.697–1.323]	0.804
***Associated symptoms***					
Cough	178 (11.3)	15 (4.5)	0.001		
Vomiting	526 (33.4)	110 (32.6)	0.778		
Abdominal pain	613 (39.0)	103 (30.6)	0.004		
Chest pain	42 (2.7)	7 (2.1)	0.532		
Syncope	140 (8.9)	48 (14.2)	0.003		
Gastrointestinal bleeding	89 (5.7)	12 (3.6)	0.116		
Asthenia	314 (20.0)	72 (21.4)	0.560		
Confusion	77 (4.9)	35 (10.4)	<0.001		
Anuresis	28 (1.8)	7 (2.1)	0.712		
***Laboratory parameters***					
Hb (g/dL)	12.4 [10.6–13.9]	11.2 [9.9–12.8]	**<0.001**		
WBC (cell/mm^3^)	9.7 [6.6–14.1]	11.1 [5.8–16.9]	**<0.001**	1.002 [0.989–1.015]	0.759
Creatinine (mg/dL)	0.9 [0.7–1.3]	1.4 [0.9–2.4]	0.360	0.943 [0.865–1.027]	0.179
Blood glucose (mg/dL)	111.0 [96.0–138.0]	116.5 [96.2–148.0]	0.090		
PCT (ng/dL)	0.14 [0.05–0.52]	2.6 [0.36–20.78]	<0.001		
PCT > 0.5 ng/dL (%)	407 (25.9)	245 (72.7)	**<0.001**	6.694 [4.869–9.202]	<0.001
CRP (mg/L)	68.4 [22.0–143.7]	148.7 [69.2–217.4]	**<0.001**	1.002 [1.000–1.003]	0.025
***Clinical history***					
Charlson comorbidity score	4 [2–6]	5 [3–7]	**<0.001**	1.060 [0.992–1.132]	0.087
Immunosuppressive therapy	41 (2.6)	13 (3.9)	0.209	2.069 [0.990–4.370]	0.067
IBD	128 (8.1)	6 (2.4)	<0.001	0.481 [0.212–1.090]	0.003
Ischemic cardiac disease	136 (8.6)	38 (11.3)	0.128		
Heart failure	101 (6.4)	22 (6.5)	0.942		
COPD	122 (7.8)	28 (8.3)	0.732		
Diabetes	190 (12.1)	47 (13.9)	0.345		
Chronic renal failure	234 (14.9)	96 (28.5)	<0.001		
Liver disease	123 (7.8)	22 (6.5)	0.417		
Dementia	122 (7.8)	25 (7.4)	0.833		
Cancer	1290 (18.4)	88 (26.)	0.001		
***Clinical outcomes***					
Abdominal surgery	384 (5.3)	17 (5.0)	0.826		
Lenght of hospitalization	8.4 [5.4–14.0]	13.5 [7.8–25.1]	<0.001		

## Data Availability

All data are included within the main test.

## References

[1] GBD 2016 Causes of Death Collaborators (2018). Estimates of the global, regional, and national morbidity, mortality, and aetiologies of diarrhoea in 195 countries: A systematic analysis for the Global Burden of Disease Study 2016. Lancet Infect. Dis..

[2] GBD 2019 Diseases and Injuries Collaborators (2020). Global burden of 369 diseases and injuries in 204 countries and territories, 1990–2019: A systematic analysis for the Global Burden of Disease Study 2019. Lancet.

[3] Bresee J.S., Marcus R., Venezia R.A., Keene W.E., Morse D., Thanassi M., Brunett P., Bulens S., Beard R.S., Dauphin L.A. (2012). The etiology of severe acute gastroenteritis among adults visiting emergency departments in the United States. J. Infect. Dis..

[4] Lai C.C., Ji D.D., Wu F.T., Mu J.J., Yang J.R., Jiang D.D., Lin W.Y., Chen W.T., Yen M.Y., Wu H.S. (2016). Etiology and Risk Factors of Acute Gastroenteritis in a Taipei Emergency Department: Clinical Features for Bacterial Gastroenteritis. J. Epidemiol..

[5] Sajeed S.M., De Dios M.P., Dan O.W.J., Punyadasa A.C. (2023). Defining a clinical prediction rule to diagnose bacterial gastroenteritis requiring empirical antibiotics in an emergency department setting: A retrospective review. Indian J. Gastroenterol..

[6] Ullah M.K., Dayam F., Ahmed A., Ahmad S., Munawar M., Jahangir S., Daftani M.H., Ali Z., Kakar B., Farooq A. (2024). A Multidisciplinary Approach in the Management of Infectious Diarrhea in the Emergency Department. Cureus.

[7] Riddle M.S., Dupont H.L., Connor B.A. (2016). ACG clinical guideline: Diagnosis, treatment, and prevention of acute diarrheal infections in adults. Am. J. Gastroenterol..

[8] Shane A.L., Mody R.K., Crump J.A., Tarr P.I., Steiner T.S., Kotloff K., Langley J.M., Wanke C., Warren C.A., Cheng A.C. (2017). Infectious Diseases Society of America clinical practice guidelines for the diagnosis and management of infectious diarrhea. Clin. Infect. Dis..

[9] Safdar N., Said A., Gangnon R.E., Maki D.G. (2002). Risk of hemolytic uremic syndrome after antibiotic treatment of *Escherichia coli* O157:H7 enteritis: A meta-analysis. JAMA.

[10] Baron E.J., Miller J.M., Weinstein M.P., Richter S.S., Gilligan P.H., Thomson R.B., Bourbeau P., Carroll K.C., Kehl S.C., Dunne W.M. (2013). A guide to utilization of the microbi- ology laboratory for diagnosis of infectious diseases: 2013 recommendations by the Infectious Diseases Society of America (IDSA) and the American Society for Microbiology (ASM). Clin. Infect. Dis..

[11] Jabak S.J., Kawam L., El Mokahal A., Sharara A.I. (2022). Management of acute diarrhea in the emergency department of a tertiary care university medical center. J. Int. Med. Res..

[12] Shastri Y.M., Bergis D., Povse N., Schäfer V., Shastri S., Weindel M., Ackermann H., Stein J. (2008). Prospective multicenter study evaluating fecal calprotectin in adult acute bacterial diarrhea. Am. J. Med..

[13] Thia K.T., Chan E.S., Ling K.L., Ng W.Y., Jacob E., Ooi C.J. (2008). Role of procalcitonin in infectious gastroenteritis and inflammatory bowel disease. Dig. Dis. Sci..

[14] Shin H.J., Kang S.H., Moon H.S., Sung J.K., Jeong H.Y., Kim J.S., Joo J.S., Lee E.S., Kim S.H., Lee B.S. (2018). Serum procalcitonin levels can be used to differentiate between inflammatory and non-inflammatory diarrhea in acute infectious diarrhea. Medicine.

[15] Cancella de Abreu M., Cassard C., Cherubini I., Houas E., Dechartres A., Hausfater P. (2023). Usefulness of serum procalcitonin and point-of-care multiplex PCR gastro-intestinal panel in acute diarrhoea or colitis in the emergency department. Biomarkers.

[16] Axelrad J.E., Freedberg D.E., Whittier S., Greendyke W., Lebwohl B., Green D.A. (2019). Impact of Gastrointestinal Panel Implementation on Health Care Utilization and Outcomes. J. Clin. Microbiol..

[17] Keske Ş., Zabun B., Aksoy K., Can F., Palaoğlu E., Ergönül Ö. (2018). Rapid Molecular Detection of Gastrointestinal Pathogens and Its Role in Antimicrobial Stewardship. J. Clin. Microbiol..

[18] Becker K.L., Nylén E.S., White J.C., Müller B., Snider R.H. (2004). Procalcitonin and the calcitonin gene family of peptides in inflammation, infection, and sepsis: A journey from calcitonin back to its precursors. J. Clin. Endocrinol. Metab..

[19] Simon L., Gauvin F., Amre D.K., Saint-Louis P., Lacroix J. (2004). Serum procalcitonin and C-reactive protein levels as markers of bacterial infection: A systematic review and meta-analysis. Clin. Infect. Dis..

[20] Limper M., de Kruif M.D., Duits A.J., Brandjes D.P., van Gorp E.C. (2010). The diagnostic role of procalcitonin and other biomarkers in discriminating infectious from noninfectious fever. J. Infect..

[21] Schuetz P., Wirz Y., Sager R., Christ-Crain M., Stolz D., Tamm M., Bouadma L., Luyt C.E., Wolff M., Chastre J. (2017). Procalcitonin to initiate or discontinue antibiotics in acute respiratory tract infections. Cochrane Database Syst. Rev..

[22] Schuetz P., Wirz Y., Sager R., Christ-Crain M., Stolz D., Tamm M., Bouadma L., Luyt C.E., Wolff M., Chastre J. (2018). Effect of procalcitonin-guided antibiotic treatment on mortality in acute respiratory infections: A patient level meta-analysis. Lancet Infect. Dis..

[23] Schuetz P., Falsey A.R. (2018). Procalcitonin in patients with fever: One approach does not fit all. Clin. Microbiol. Infect..

[24] Covino M., Gallo A., Montalto M., De Matteis G., Burzo M.L., Simeoni B., Murri R., Candelli M., Ojetti V., Franceschi F. (2021). The Role of Early Procalcitonin Determination in the Emergency Departiment in Adults Hospitalized with Fever. Medicina.

[25] Huang D.T., Yealy D.M., Filbin M.R., Brown A.M., Chang C.H., Doi Y., Donnino M.W., Fine J., Fine M.J., Fischer M.A. (2018). ProACT Investigators. Procalcitonin-Guided Use of Antibiotics for Lower Respiratory Tract Infection. N. Engl. J. Med..

[26] Covino M., Manno A., Merra G., Simeoni B., Piccioni A., Carbone L., Forte E., Ojetti V., Franceschi F., Murri R. (2020). Reduced utility of early procalcitonin and blood culture determination in patients with febrile urinary tract infections in the emergency department. Intern. Emerg. Med..

[27] Charlson M.E., Pompei P., Ales K.L., MacKenzie C.R. (1987). A new method of classifying prognostic comorbidity in longitudinal studies: Development and validation. J. Chronic Dis..

[28] Jetté N., Quan H., Hemmelgarn B., Drosler S., Maass C., Moskal L., Paoin W., Sundararajan V., Gao S., Jakob R. (2010). The development, evolution, and modifications of ICD-10: Challenges to the international comparability of morbidity data. Med. Care.

[29] Karras D.J., Ong S., Moran G.J., Nakase J., Kuehnert M.J., Jarvis W.R., Talan D.A., EMERGEncy ID NET Study Group (2003). Antibiotic use for emergency department patients with acute diarrhea: Prescribing practices, patient expectations, and patient satisfaction. Ann. Emerg. Med..

[30] Ismaili-Jaha V., Shala M., Azemi M., Spahiu S., Hoxha T., Avdiu M., Spahiu L. (2014). Sensitivity and specificity of procalcitonin to determine etiology of diarrhea in children younger than 5 years. Mater. Sociomed..

[31] Al-Asy H.M., Gamal R.M., Albaset A.M.A., Elsanosy M.G., Mabrouk M.M. (2017). New diagnostic biomarker in acute diarrhea due to bacterial infection in children. Int. J. Pediatr. Adolesc. Med..

[32] Hanlon P., Nicholl B.I., Jani B.D., Lee D., McQueenie R., Mair F.S. (2018). Frailty and pre-frailty in middle-aged and older adults and its association with multimorbidity and mortality: A prospective analysis of 493 737 UK Biobank participants. Lancet Public. Health.

[33] Ward D.D., Ranson J.M., Wallace L.M.K., Llewellyn D.J., Rockwood K. (2022). Frailty, lifestyle, genetics and dementia risk. J. Neurol. Neurosurg. Psychiatry.

[34] Liu X., Wang Y., Shen L., Sun Y., Zeng B., Zhu B., Dai F. (2023). Association between frailty and chronic constipation and chronic diarrhea among American older adults: National Health and Nutrition Examination Survey. BMC Geriatr..

[35] Peng F., Chang W., Xie J.F., Sun Q., Qiu H.B., Yang Y. (2019). Ineffectiveness of procalcitonin-guided antibiotic therapy in severely critically ill patients: A meta-analysis. Int. J. Infect. Dis..

[36] Elsing C., Ernst S., Kayali N., Stremmel W., Harenberg S. (2011). Lipopolysaccharide binding protein, interleukin-6 and C-reactive protein in acute gastrointestinal infections: Value as biomarkers to reduce unnecessary antibiotic therapy. Infection.

